# Case Report: Primary malignant mesothelioma of the left atrium easily misdiagnosed as myxoma

**DOI:** 10.3389/fcvm.2024.1398311

**Published:** 2024-06-13

**Authors:** Shuai Luo, Yao Li, Jin Li, Jiafei Zeng, Jinjing Wang

**Affiliations:** Department of Pathology, Affiliated Hospital of Zunyi Medical University, Zunyi, Guizhou, China

**Keywords:** left atrium, malignant mesothelioma, diagnosis, differential diagnosis, myxoma

## Abstract

**Background:**

Malignant mesothelioma (MM) is a rare and aggressive tumor that is found in the pleura and peritoneum. A few cases of MM in the pericardium and tunica vaginalis testis have been reported. Moreover, primary occurrence in the atrium is extremely rare. The visual appearance of this tumor is similar to that of a common atrial myxoma, which makes it challenging for clinicians and radiologists to diagnose and treat this disease.

**Case demonstration:**

An 18-year-old woman presented with symptoms of chest pain, shortness of breath, cough, and expectoration for 7 days. Echocardiography was performed on the patient, which revealed an atrial mass. Myxoma was one of the differential diagnoses. The tumor was an elliptical mass with tips, and the cut surface was jelly-like, similar to myxoma. After surgery, a pathologic examination of the biopsied tumor confirmed epithelial-type MM. During postoperative follow-up, no recurrence of the tumor was observed.

**Conclusions:**

MM originating in the atrium is considered to be extremely rare. Consequently, clinicians can easily misdiagnose atrial MM as a myxoma. Moreover, to confirm the diagnosis, histopathologic biopsy, histomorphological characterization, immunohistochemistry, and molecular genetic testing are required. Therefore, clinical diagnosis and treatment of MM are challenging.

## Background

Malignant mesothelioma (MM) is characterized as a tumor with an epithelial–mesenchymal transition. The pleura is reported as the most common site of occurrence, accounting for 90% of patient cases. Other sites of occurrence include the peritoneum and pericardium. In a few cases, the tunica vaginalis testis has been reported as the site of occurrence. The atrium is considered an extremely rare primary site for MM. The histomorphology of MM has been observed to be varied, and familiarity with the histologic pattern of MM is required for making any diagnosis or differential diagnosis. In this study, a case report of a patient with MM diagnosed at our hospital is presented. In addition, the relevant literature is reviewed to analyze the pathomorphological characteristics and clinical features, with the aim of improving clinicians’ understanding of MM.

## Case demonstration

**Figure 1:**
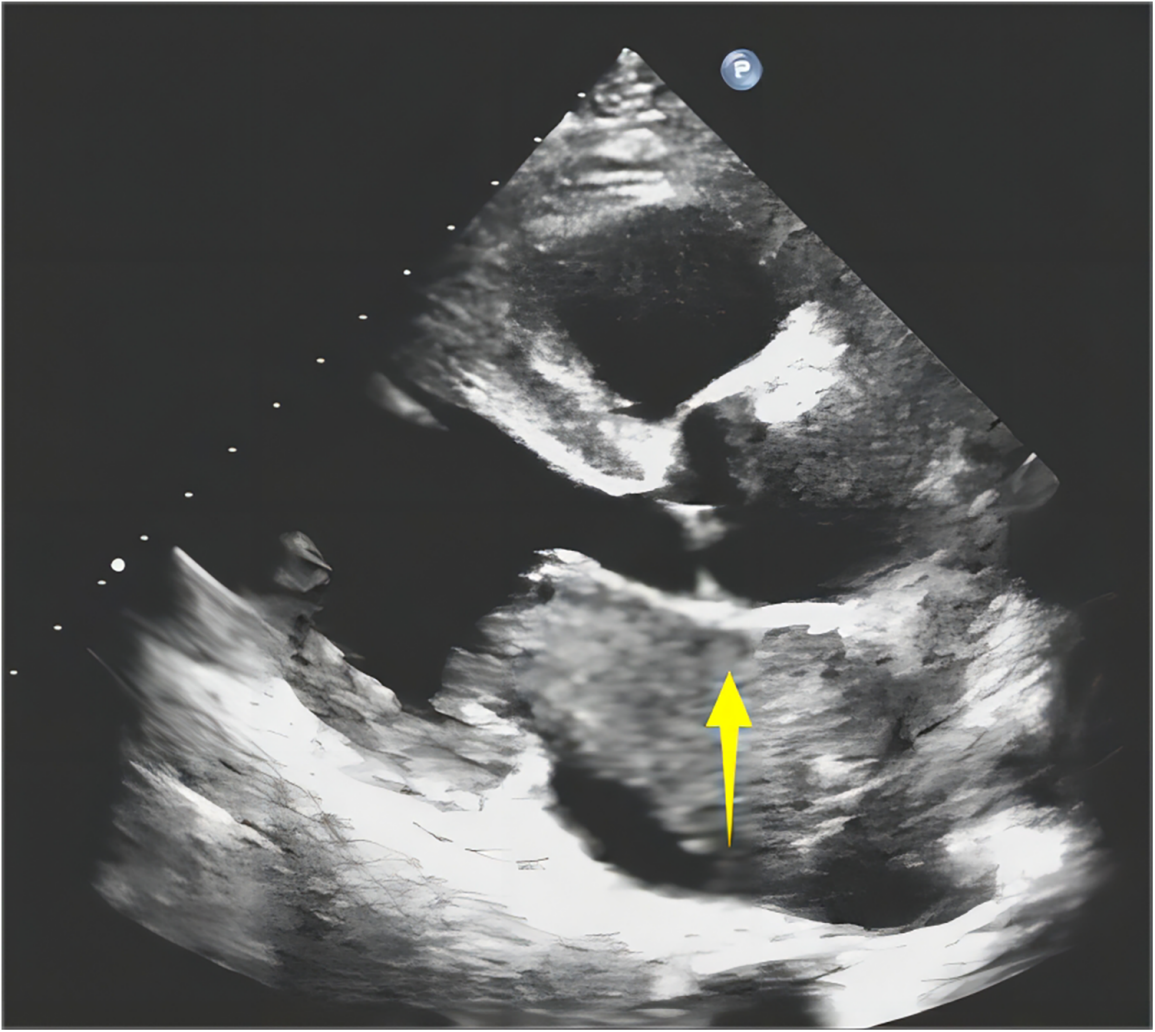
Ultrasonography shows a left atrial isoechoic mass (↑).

An 18-year-old woman presented with symptoms of chest pain and shortness of breath after activity. She had a cough and expectoration for 7 days because of an infection in the upper respiratory tract. Upon admission, a physical examination was conducted, which showed a symmetrical thorax without malformation and symmetrical respiratory movement in both lungs. On auscultation, coarse respiratory sounds were heard, and wet rales were detected at the bottom of both lungs. Her blood pressure was 113/50 mmHg. The heart rate was 115 beats/min. Echocardiography revealed echo groups in the left atrium, obstruction and mild regurgitation of the mitral valve, moderate regurgitation of the tricuspid valve, and mild regurgitation of the valve (Figure 1). Left atrial myxoma was considered a differential diagnosis. There was no uplift or depression in the precardiac area, and the heart boundary was enlarged to the left lower region. The rhythm was steady, and diastolic murmurs were audible in the apical area. The patient had no history of trauma or surgery, and no other significant findings were noted. The patient underwent tumor resection under direct intracardiac visualization with the use of general anesthesia and extracorporeal circulation. During surgery, the mass was found in the left atrium. It was endogenous, with poor mobility. The tip of the mass was attached to the atrial septum. The rest of the right atrium and ventricle did not have a clear mass.

**Figure 2:**
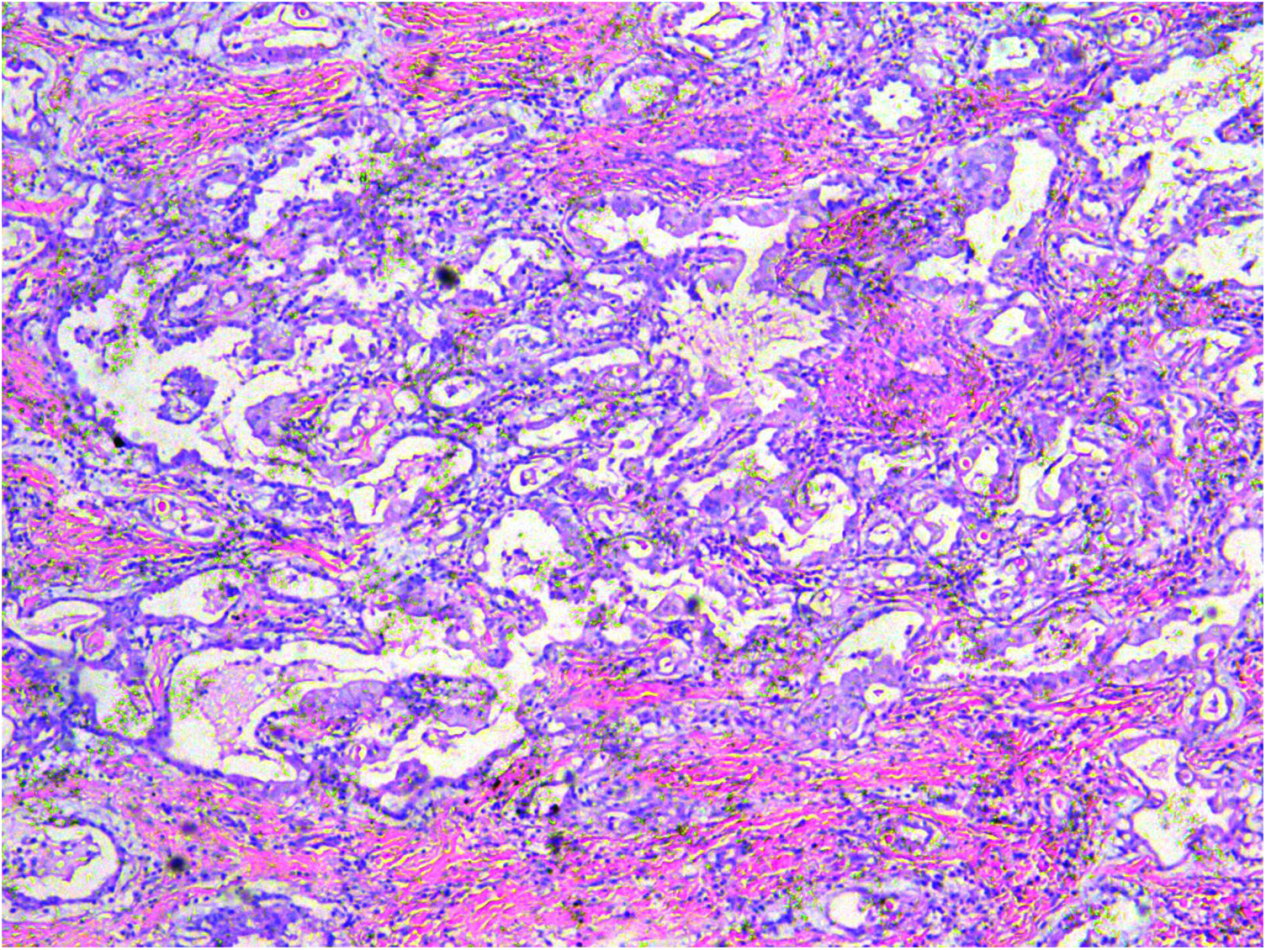
A microcystic or gridded arrangement, similar to adenomatoid tumors, H&E ×100.

**Figure 3:**
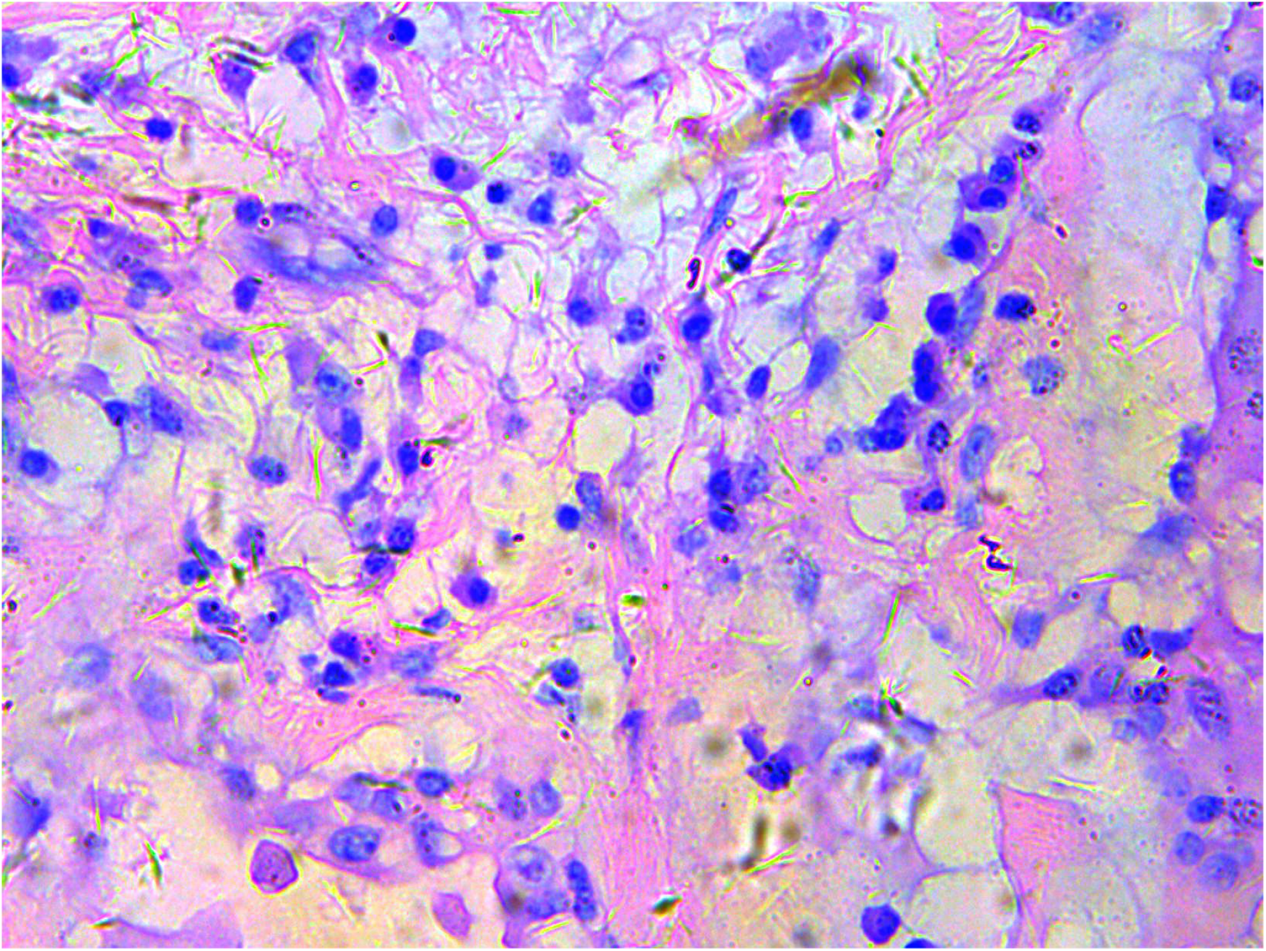
Epithelioid cells are vacuolar or cubic, with acidophilic and abundant cytoplasm and large deeply stained nuclei; H&E ×200.

**Figure 4:**
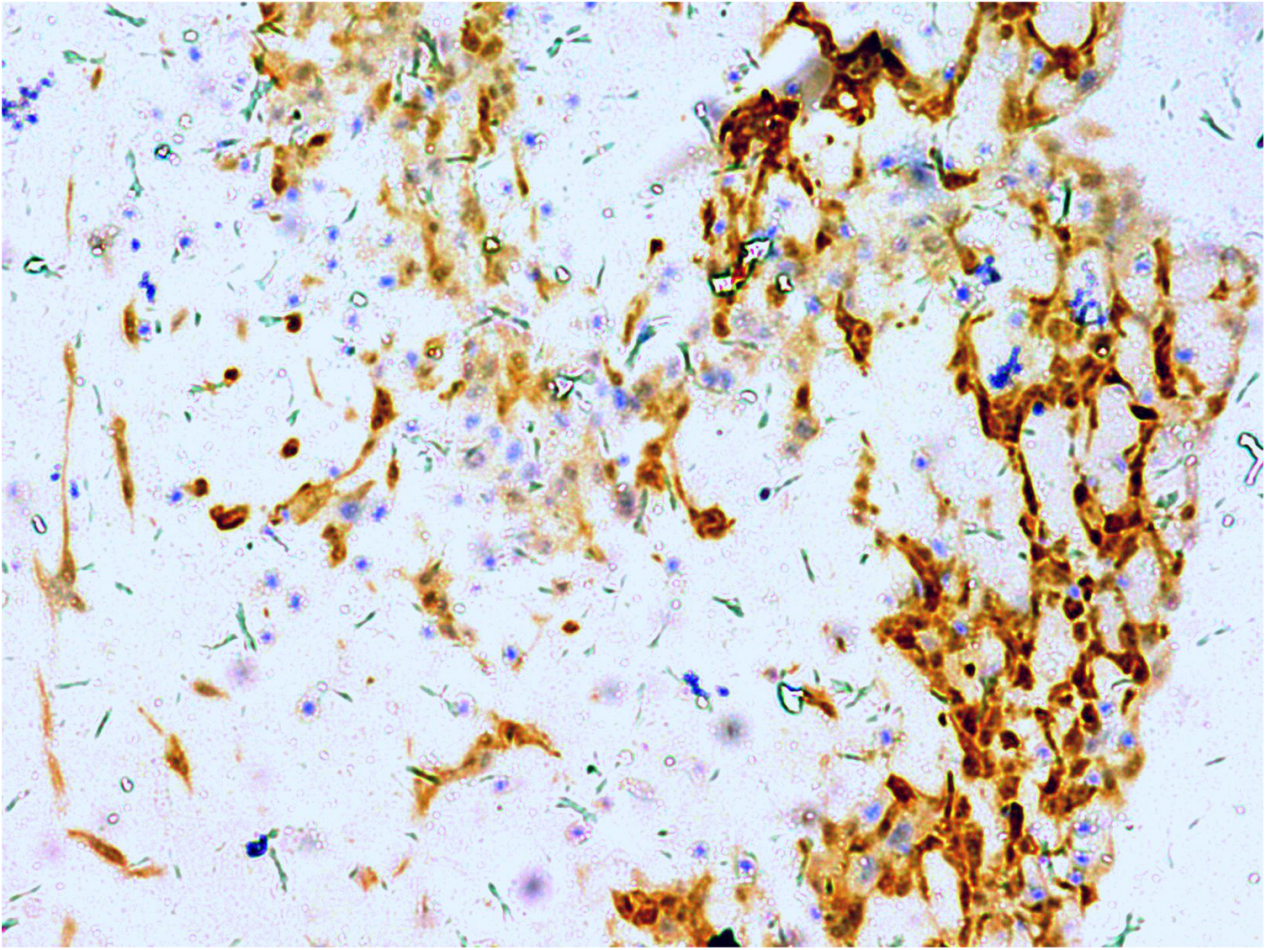
Calretinin is positive for diffuse cell membrane; EnVision, ×200.

A gross pathological examination indicated a grayish-white, jelly-like mass with a size of 5 cm × 5 cm × 4 cm, and the tip was approximately 5 mm. No envelope was seen. Microscopic examination identified numerous fissures in the tumor tissue. Glandular ducts and microcystic structures were seen (Figure 2). The tumor stroma and mucosa were fibrous; the cells were epithelioid and either vacuolated or cuboidal. The cytoplasm was acidophilic and abundant; the nucleus was large and deeply stained. The karyoplasmic index was high, and the nucleolus was clearly visible (Figure 3). Immunohistochemistry showed calretinin (Figure 4), and CK5/6, CK, D2-40, WT-1, and vimentin were positively expressed. TTF-1, napsin A, CEA, CK7, CD31, CD34, and other markers were negative. The percentage of the Ki-67 positive index was approximately 40.

Based on the histological and immunohistochemical results, the pathologic diagnosis was confirmed as left atrial epithelioid MM.

The patient did not receive postoperative radiotherapy, and no recurrence was seen on repeat echocardiography after 3 months.

## Discussion

MM is a rare and aggressive tumor occurring in the pleura and peritoneum. In a few patient cases, the pericardium, tunica vaginalis testis, and female reproductive organ system have been reported as the sites of occurrence (1). MM is mainly diagnosed in patients over the age of 60 and is found to have a higher prevalence in men. The majority of cases of mesothelioma are attributed to occupational asbestos exposure (2). However, other causes of mesothelioma are associated with exposure to erionite, simian vacuolating virus 40 (SV40) infection, radiation, chronic inflammation, and genetic susceptibility (3). Nevertheless, recent studies have reported that younger female patients less exposed to asbestos have a higher frequency of prior radiotherapy and a higher family history of breast cancer than older patients (4). The patient reported in this study was an 18-year-old woman with no history of asbestos exposure or tumors. The tumor was confined within the left atrium without pleural involvement. This is an extremely rare occurrence of tumors and has been reported in only a few cases (5).

The origin of MM is not fully understood, but in our patient, the tumor was found to have originated from mesothelial cells. Studies have demonstrated that the tumor may originate from subcellular mesothelial cells, which can differentiate in various directions (6). Clinical manifestations depend on the location of the tumor. Furthermore, when the heart is the primary site, specific manifestations are not typically observed. Patients are admitted to the hospital with chest pain and shortness of breath after physical activity. Upon ultrasonographic examination, a strong echo with a luminescent cluster is observed, within which echogenic areas of varying size can be detected. The boundaries of these findings are not well defined. These ultrasonographic findings bear a resemblance to those of cardiac tumors, myxomas, and other conditions (7). Therefore, MM can be commonly misdiagnosed based on clinical manifestations and imaging results. Accurate diagnosis should be based on a combination of histopathology, immunohistochemistry, and molecular examinations.

MM is classified as a heterogeneous tumor based on its histomorphology. According to its histologic morphology, it is categorized into three subtypes: epithelioid, sarcomatoid, and biphasic (8). Among these subtypes, epithelioid mesothelioma is considered to be more prevalent than sarcomatoid and biphasic forms.

The most common morphologic variants of epithelioid mesothelioma are tubular, papillary, solid, and trabecular. Psammoma bodies may appear in any of these variants. Other uncommon variants include micropapillary, adenomatoid (microcystic), clear cell, migratory, metaplastic, small cell, and lymphohistiocytic (8, 9). Generally, epithelioid tumors contain polygonal, vacuolated, ovoid, or cuboidal cells that mimic reactive mesothelial cells and respond to various types of injury. However, a poorly differentiated epithelioid tumor with pathological mitotic signs was seen in our patient (10).

Sarcomatoid mesothelioma is the least common but most aggressive of the three histologic types of mesothelioma (11). The sarcomatoid types are usually characterized by a fascicular or irregularly arranged proliferation of spindle cells with varying degrees of nuclear atypia and mitotic figures. Sarcomatoid tissues rarely have heterologous differentiation, such as osteoid, bone, or cartilage (8, 12). The fibrous reactive mesenchyme in epithelioid mesothelioma may be sparse or prominent with varying degrees of cellularity, which makes it challenging to distinguish it from a true sarcomatoid component. In these cases, immunohistochemistry may be helpful in showing the presence of BRCA1-associated protein 1 (BAP-1) and demonstrating a lack of expression in the sarcomatoid mesothelioma region (13).

Biphasic MM is characterized by the presence of mixed tumors consisting of epithelioid and sarcomatoid types, with each type constituting at least 10% of the tumors (8). In our patient, we reported an epithelioid malignant MM with vacuolated or cuboidal tumor cells arranged in microcystic or lattice-like patterns, resembling an adenomatoid tumor.

Immunohistochemical markers play a crucial role in histologic and differential diagnoses. However, it has been observed that no antibody can achieve 100% sensitivity and 100% specificity. Consequently, the Guidelines for the Diagnosis and Treatment of Malignant Pleural Mesothelioma recommend combining immunohistochemical markers with at least two positive and two negative markers to diagnose MM (1). Antibodies that can detect mesothelioma expression markers, including calretinin, WT1, D2-40, and CK5/6, were chosen. Immunohistochemical indices including CEA, TTF-1, Napsin A, MOC31, Ber-EP4, and BAP1 were not expressed (8, 10). P53 has also been reported to be often abnormally expressed in MM, which can be distinguished from reactive mesothelial hyperplasia. SOX6, as a new immunohistochemical marker of MM, has similar sensitivity to CR and D240 in differentiating epithelial MPM from lung adenocarcinoma, but it has better specificity. In addition, studies have shown that the expression of SOX6 was more sensitive than that of WT-1 (8, 10).

It has been demonstrated through studies (6) that partial loss of 1p21M-22, 3p14–25, 4q, 6q, 9p21, 13q13–14, 14q, and haploid chromosome 22, along with repetitive loss of 17p12-pter, are associated with molecular genetic changes in MM. Fluorescence *in situ* hybridization has revealed that deletion of the p16 gene is observed in approximately 35% of MM cases.

(1) During diagnosis, various primary and secondary tumors must be differentiated from MM. In the case presented here, the tumor growth site was rare, and the histological morphology was non-specific. Consequently, the possibility of misdiagnosis is high, and certain diseases listed here need to be ruled out.
(2) Reactive mesothelial hyperplasia: Although reactive mesothelial hyperplasia may show atypia, it is not significant. There are no tubules and no papillae formation, which would suggest MM. Furthermore, the most reliable morphological criterion for MM proliferation is a true infiltration of the mesenchyme (14). Currently, BAP-1 immunohistochemistry and p16 fluorescent *in situ* hybridization are the most effective analytical methods to identify benign and malignant mesothelial lesions (13, 15, 16). Studies have demonstrated that the lack of BAP-1 and p16 expression in MMs contributes to benign and malignant mesothelial cell proliferation.
(3) Myxoma in the atrium is the most common type of primary atrial tumor, and hence, it is common to misdiagnose MM as myxoma. Further, myxoma can also express CK and calretinin, which needs to be combined with the histopathology of myxoma (the whole tumor shows a large number of mucoid matrix and scattered tumor cells in the form of strips, networks, or single arrangements). Some of them can be arranged around the blood vessels, which will lead to the formation of a perivascular ring and will be often accompanied by scattered lymphocyte and plasma cell infiltration, bleeding, and hemosiderin deposition. The tumor may present with secondary changes, including fibrosis, cystic change, necrosis, thrombosis, calcification, ossification and Gamna-Gandy body. Myxoma immunohistochemistry demonstrates positivity for S100, CD34 and CD31, while it is negative for markers such as WT1, D2-40 and CK5/6. In terms of molecular genetics, approximately two-thirds of atrial myxomas exhibit mutations in the PRKAR1A gene (7).
(4) Atrial fibrosarcoma: This is an extremely rare and pure malignant spindle cell mesothelioma. The histologic morphology is similar to that of fibrosarcoma. However, the longitudinal and transverse cell bundles are not as obvious as in fibrosarcoma. The migratory components between epithelial mesothelial cells and spindle cells can be seen in the tumor tissues after a careful examination. The expression of CK and calretinin can be differentiated from that of fibrosarcoma.
(5) Synovial sarcoma is characterized by a histological pattern similar to that of MM, and biphasic differentiation can be achieved. The epithelioid component can be a synovial-like cuboidal epithelium or adenoidal columnar epithelium. The sarcomatoid component cells can be fibroblast-like and perivascular-like. In addition, biphasic expression is often observed in immunohistochemistry, with calretinin being focally positive. However, an important point differentiating synovial sarcoma from MM is negative WT1, with a t(X; 18) or SS18-SSX gene fusion (17). Synovial sarcomas are most commonly found in the extremities and rarely metastasize to the atrium.
(6) Metastatic adenocarcinoma can be challenging to differentiate from malignant epithelioid MM when the latter presents with only glandular and adenoid structures without malignant spindle cells. However, adenocarcinoma is generally positive for CEA, TTF-1, and napsin A and negative for calretinin and vimentin, whereas in MM, the scenario is reversed (18). If estrogen receptor, progesterone receptor, and gross cystic disease fluid protein 15 (GCDFP-15) expressions are positive, metastatic breast cancer is considered (19). In the case of the patient in this study, no tumor was seen at any other site, and the patient had no history of tumor.

## Treatment and prognosis

The prognosis for MM is poor. The average survival time of a patient usually does not exceed 12 months (20). There is currently no standard and effective treatment available, and complete surgical resection is usually the mainstay of therapy. However, it is sometimes challenging to achieve complete surgical resection. Our patient was followed up for 3 months, following which she had no tumor recurrence or metastasis. Pemetrexed/cisplatin combination chemotherapy has been widely used in treating MM. However, the efficacy rate after chemotherapy ranges between 30% and 40%, and the long-term survival rate remains poor (21). With the advent of next-generation sequencing technology, such as BAP-1 (13), neurofibromatosis type 2 (22), and cyclin-dependent kinase inhibitor 2A (p16/CDKN2A) (23), other gene therapies are currently under investigation. Therefore, differential diagnosis and treatment are based on genetic data that require further research.

## Data Availability

The original contributions presented in the study are included in the article/Supplementary Material, further inquiries can be directed to the corresponding author.
